# Impacts of Rotor Design, Screw Design, and Processing Parameters in a Farrel Continuous Mixer

**DOI:** 10.3390/polym17050619

**Published:** 2025-02-25

**Authors:** Mansour Alotaibi, Carol Forance Barry

**Affiliations:** Department of Plastics Engineering, University of Massachusetts Lowell, Lowell, MA 01879, USA; mansour_alotaibi@student.uml.edu

**Keywords:** continuous mixer, rotor design, screw design, melt compounding

## Abstract

Continuous mixers, which consist of a section with non-intermeshing counter-rotating rotors and a single-screw extruder, were developed for thermoset rubber and are often used for compounding of heavily filled thermoplastics. Due to the high mixing efficiency and tight control of shear levels, they may be suited for other compounding other material systems. Little work, however, has been reported on compounding with these mixers, and preliminary work with polypropylene showed interesting limitations of the mixing parameters. Therefore, this study investigated the effects of nine rotor designs, two single-screw designs, rotor speed, feed rate, and orifice setting on the residence time and melt temperature in a Farrel Compact Processor. In general, single-stage rotors produced lower mixer residence times and melt temperatures compared to longer two-stage and high dispersion rotors. Higher rotor speeds and feed rates and smaller orifice openings generally reduced mixer residence times. Higher rotor speeds increased mixer melt temperatures, whereas higher feed rates and smaller orifice openings produced lower mixer melt temperatures. The single-screw design impacted the residence time but not the melt temperature. Overall, the results of this work provided strategies for optimizing the processing parameters and rotor design selection when melt compounding with continuous mixers.

## 1. Introduction

With thermoplastics, melt compounding is an essential step after polymer production due to low bulk density and the formation of dust in powders from the reactors and for incorporating additives such as antioxidants to produce polymer pellets [1]. Moreover, tailored and specialty thermoplastics can be manufactured by melt compounding multiple components to create polymers blends, polymers with fillers and/or fibers, and reactive extrusions [1]. For commercial melt compounding, the most commonly used system is a co-rotating twin screw extruder [2]. Due to the flexibility in the screw configuration, full-intermeshing co-rotating twin screw extruders produce excellent distributive and dispersive mixing of a wide range of materials and are capable of degassing the air and volatile chemicals present in polymer melts. While these extruders can achieve high throughput due to their efficient design, the applications of these extruders are generally volume limited by the intake capacity of the screws, are torque-limited by the capacity of the drives, and have poor pumping efficiency due to low discharge pressures [1]. With co-rotating twin screw extruders, strategies to overcome these limitations include changing the screw design and incorporating a gear pump downstream of the extruder. Another approach to the low pumping capacity, however, is using alternative compounding equipment with higher free volume, including extruders with three, four, and eight screws and continuous mixers [1,3,4,5].

A continuous mixer is a two-stage compounding system consisting of mixing unit with two counter-rotating, non-intermeshing rotors followed by a single-screw extruder [6]. Melting and mixing of the material are typically performed in the mixer unit; in some applications, the extruder can help to homogenize the melt further or degas the final product [1]. These rotors impart moderate pressure levels; therefore, the polymer melt produced in the mixer exits the mixer unit and is then passed either through a single-screw extruder or a gear pump before the compound is pelletized [6]. Although the continuous mixer was originally intended to replace the batch mixers for compounding rubber and carbon black [7], it had limited use because it can only process powdered or granular rubbers [8,9]. Currently, it is widely used for melt compounding high levels of additives into thermoplastics because it can perform melting, dispersive and distributive mixing, and degassing. Compared to co-rotating twin screw extruders, continuous mixers employ lower processing temperatures, lower mechanical shear, and lower specific energies. They also can handle mineral filler levels up to 80 wt.% [7].

**Figure 1:**
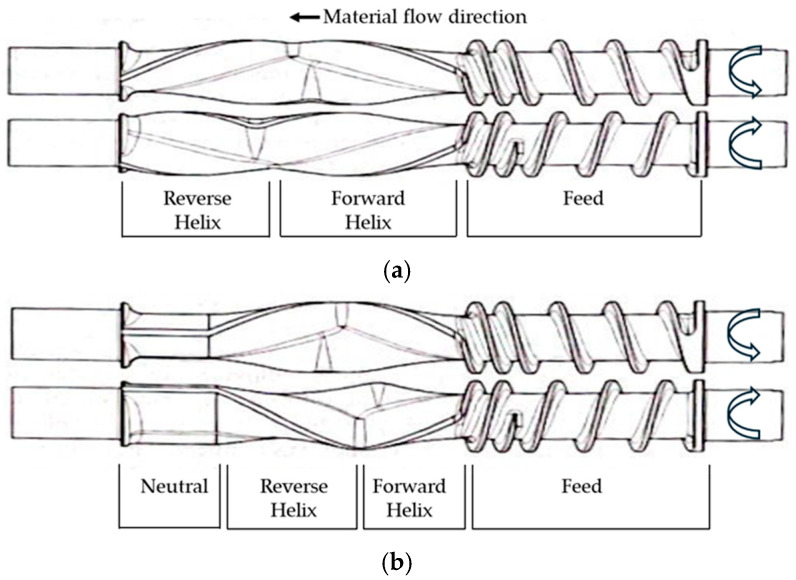
Two standard rotor designs: (**a**) style 7 (7L/7R); (**b**) style 15 (15L/15R).

With continuous mixers, mixing and melting quality are directly related to the rotor design. As shown in Figure 1, standard rotors contain two sections: one for feeding and one for mixing. The feeding sections have cylindrical designs similar to those in counter-rotating twin screw extruders. The mixing section begins with a forward helix shape section and is followed by a reverse helix section. The primary function of the forward-helix section is to convey the material forward while compacting, heating, and beginning softening and melting of the solids. The reverse-helix section creates back flow, which improves melting and provides more intensive mixing [1]. The rotation of the two rotors provides the thermal energy required for melting by friction between the solid particles and metal walls, inter-particle friction, and viscous dissipation. The mixing intensity is mainly based on the location and length of the apex zone, which is the intersection area between the forward and reverse-helix sections, because it has the highest fill and the highest shear area in the mixer unit. Consequently, it develops the greatest stresses, resulting in the highest mixing intensity [10]. Neutral sections, which are present in style 15 rotor (Figure 1b), help pump the material through the orifice.

The processing variables of the continuous mixer have been evaluated for thermoplastics. Galle and White [11] studied the residence time distribution, melting, and melting homogeneity of polypropylene in a continuous mixer (Kobe Steel, model: Nex-T) using a style 15 rotor design (Figure 1b); the processing variables were the feed rate, rotor speed, screw speed, orifice position, and mixing chamber temperature. With increasing feed rate, rotor speed, screw speed, and orifice closing, a significant increase in melt temperature was observed. Residence time decreased with an increase in feed rate, rotor speed, screw speed, and orifice closing. Additionally, the melt homogeneity improved with a lower feed rate, higher rotor speed, and a smaller orifice opening. Chen et al. [12] explored the effects of rotor speed (between 500 and 800 rpm), feed rate (between 1.58 and 4.28 kg/h), and orifice position (between 25 and 75%) on the microstructure of high-density polyethylene (HDPE) and calcium carbonate (CaCO_3_) composites using a two-stage rotor in continuous mixer. They found that optimal deagglomeration and surface coating of the CaCO_3_ particles occurred at a rotor speed of 700 rpm, whereas higher feed rates resulted in poorer dispersion due to a shorter residence time in the mixer.

The performance of continuous mixers has been compared with the performance of other melt compounding systems. When Lahmann and Knowlton [7] evaluated mixing of polylactic acid (PLA) with different levels of talc filler using a laboratory scale continuous mixer (Farrel, model: CPeX^®^ with a style 7/15 standard rotor design) and a co-rotating twin-screw extruder, they found that the continuous mixer provided higher molecular weight retention and lower melt temperature increases compared to a co-rotating twin screw extruder; this performance was attributed to the lower processing temperatures and lower shear levels in the continuous mixer. The continuous mixer also produced dispersion that was equivalent to or better than that provided by the twin screw extruder. In contrast, Chang and White [13] investigated the reactive extrusion (maleation) of polypropylene using a fully intermeshing co-rotating twin-screw extruder, a fully intermeshing counter-rotating twin-screw extruder, a non-intermeshing counterrotating twin-screw extruder, and a continuous mixer (Kobelco, model: Nex-T). The highest level of grafted maleic anhydride was achieved when using the continuous mixer, followed by the non-intermeshing counter-rotating twin-screw extruder, the co-rotating twin screw extruder, and the fully intermeshing counter-rotating twin screw extruder. The continuous mixer exhibited a longer mean residence time and a wider residence time distribution; therefore, the maleic anhydride grafted onto the polypropylene was more effective and mixing efficiency and exposure to reactive species were enhanced. Moreover, Shon et al. [14] compared four melt compounding systems (single kneading screw extruder—i.e., Buss Kneader, co-rotating twin screw extruder, full-intermeshing counter-rotating twin screw extruder, and continuous mixer (Kobelco NEX-T)) in developing an immiscible polymer blend (75/25-polypropylene/polyamide 6). When the blends were mixed using a fixed screw speed and temperature profile, the continuous mixer produced smaller polyamide 6 droplet sizes than the other mixers with less aggressive screw designs. If the other mixers had aggressive screw designs, however, the continuous mixer produced the largest droplet sizes. The main conclusion was that the domain size was impacted by mixer type and screw design.

Each prior study with a continuous mixer has shown different residence times, residence time distributions, and shear stress levels. The residence time is the total time a given particle spends in the continuous mixer from the feed throat until it exits the die, whereas the residence time distribution is defined as histories of the material particles inside the mixer [15]. Although longer residence times can improve melting, mixing quality, and efficiency in reactive extrusion, they can cause thermal degradation during processing, particularly for heat sensitive material such as biodegradable polymers and polyethylene terephthalate (PET) [3,7,16,17,18]. The level of shear stress can elevate the melt temperature, which can reduce melt viscosity and contribute to polymer degradation. These crucial factors can influence the melting, mixing, and reaction efficiency. Therefore, each set of parameters in prior work has produced different melting and mixing quality due to the different types of flow inside the mixer.

While residence time, shear stress, and temperature have been widely investigated for co-rotating twin screw extruders [19,20], single-screw extruders [21,22], and injection molding machines [23,24], limited studies have been reported for continuous mixers. Most reported studies employed one rotor design [7,11,12,13,14], resulting in a lack of understanding about the impacts of rotor design, single-screw design, and processing parameters on the processing responses of continuous mixers. Commercial compounding with continuous mixers also relies heavily on one rotor design (style 15). Moreover, the impact of the single screw associated with the continuous mixer has not been reported. Therefore, a full factorial experimental design was performed to evaluate the effects of processing variables on the residence time and melt temperature in a laboratory continuous mixer (Farrel Pomini, mode1: CPeX, Ansonia, CT, USA). The processing variables were the feed rate, rotor design, rotor speed, orifice position, and single-screw design. This residence time and melt temperature information will enable the use of the continuous mixer with a wider range of materials.

## 2. Materials and Methods

### 2.1. Materials

A commercial grade of high-density polyethylene (HDPE) supplied by Chevron Phillips Chemical (Marlex^®^ 9018, The Woodlands, TX, USA) was used for this study. The reported melt flow index was 20 dg/min (190 °C/2.16 kg). HDPE was chosen for its excellent thermal stability, i.e., to limit thermomechanical degradation during processing. The higher melt index grade enabled evaluation of a range of rotor designs using the same barrel temperatures, rotor speeds, feed rates, and orifice settings. Preliminary work (unpublished) showed that only one rotor design (15L/15R) could process a low melt flow index (1.2 dg/min) polyolefin; this finding was consistent with industry contacts’ experiences. High melt viscosity resins compounded with other rotors often require dams to assist with melting [6], and dams are outside the scope of this study.

### 2.2. Continuous Mixer Trials

**Figure 2:**
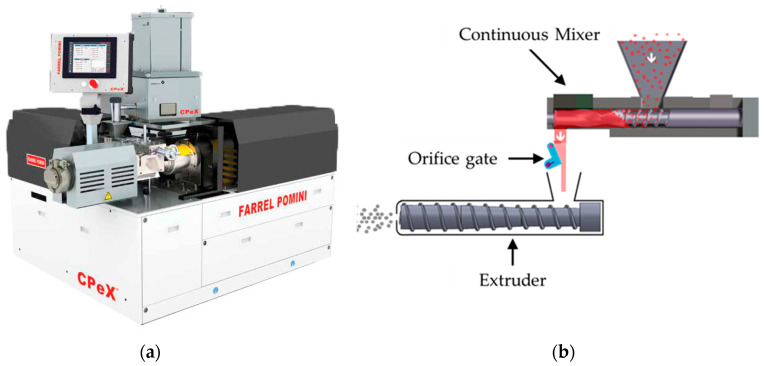
Farrel Pomini Laboratory Compact Processor (CPeX): (**a**) compounding system with the mixing section at 90° to the single-screw extruder; (**b**) schematic showing the resin flow from the feeder to the mixer, through the orifice gate, and through the single-screw extruder.

The processing of HDPE was conducted using a Farrel Pomini Laboratory Compact Processor (model: CPeX^®^, Ansonia, CT, USA) (Figure 2a). As shown in Figure 2b, this melt compounding equipment consisted of a mixing chamber with an adjustable gate orifice and single-screw extruder located downstream of the mixing chamber. The mixing section had a 35 mm bore diameter barrel and 33 mm-diameter rotors. The hot fed extruder had a 60 mm diameter single screw with a length-to-diameter ratio (L/D) of 11:1. The HDPE pellets were starved-fed to the mixing chamber using a volumetric feeder (Brabender Technologie, model FV18-0 V0L, Mississauga, ON, Canada). Melt exited the single-screw extruder through a triple strand die; the extruded strands were cooled in a water bath and then pelletized using a pelletizer (Bay Plastics Machinery Company LLC, Bay City, MI, USA).

**Figure 3:**
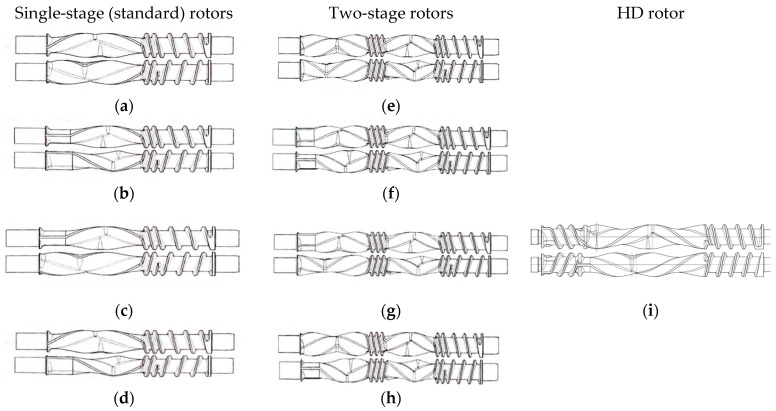
Rotor designs used in experimental trials: (**a**) style 7 (7L/7R std), (**b**) style 15 (15L/15R std), combination of styles 7 and 15 (**c**) 15L/7R std and (**d**) 7L/15R std, (**e**) two-stage style 7 (7L/7R XL), (**f**) two-stage style 15 (15L/15R XL), combination of styles 7 and 15 (**g**) 15L/7R XL and (**h**) 7L/15R XL, and (**i**) HD rotor (15L/7R HD).

The rotor designs investigated in this study are presented in Figure 3. The rotor designs included four single-stage standard (std) rotors, four two-stage (XL) rotors, and a new high dispersion (HD) rotor. The single-stage rotors had two sections, one for conveying and one for mixing; the length of the rotors was 205 mm, whereas the mixing section length was 122 mm. The two-stage rotors had two feed sections, a pre-mixing section, and a mixing section. The two-stage rotors had a rotor length of 328 mm, but the length of the mixing section also was 122 mm. The HD rotors had the same length as the two-stage rotors, but they had a longer mixing section (201 mm) and two conveying sections. Due to the longer mixing section of the HD rotor, the material spends more time in the semi-molten state [25]. The second conveying section assisted with movement of the polymer through the mixing section. For each standard and XL rotor type, there were different designs or styles. In style 7 (Figure 3a,e), the rotors end with a mixing section, whereas with style 15 (Figure 3b,f), the rotor ends with a neutral section. The apex zone, the transition between the forward helix and the reverse helix, is a critical zone in the mixer. Due to its nature, this region is usually the highest filled and the most back-mixed area in the mixer; it also covers the largest area and has the longest residence time [1]. In style 7, the apex is located further downstream than with style 15. Therefore, the mixing is less aggressive because the material spends less time as a melt; and style 7 is recommended for heat-sensitive materials. Since style 15’s apex zone is further upstream, the mixing is more aggressive as the material spends more time as a melt. Combinations of style 7 and style 15 rotors also were investigated (Figure 3c,d,g,h). Since the most widely used rotor is style 15, it was considered as the reference rotor for statistical analysis.

**Figure 4:**
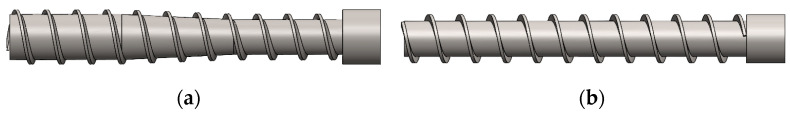
Single-screw designs used in experimental trials: (**a**) SS1 (CR = 2.3:1); (**b**) SS2 (CR = 1:1).

Figure 4 presents the two screw designs for the single-screw extruder located after the mixer. Both screws had a length-to-diameter ratio (L/D) of 11:1 and a channel depth in feed zone of 12.4 mm. Single screw 1 (SS1) had a compression ratio (CR) of 2.3:1 whereas single screw 2 (SS2) had a compression ratio of 1:1. Lower compression ratios reduce the stress on the melt [26].

As shown in Table 1, the set temperatures were held constant for all standard (std) and two stage rotor designs and extruder screw designs. Given that the single screws were pumping melt, they did not require the increasing temperature profile typical for single screws that also must melt the polymer. The target applications also tend to require one set temperature for the mixer. For example, biodegradable polymers exhibit less degradation when processed at low temperatures [3]. The melting location with the high dispersion (HD) rotor design, however, was further upstream than with standard and two-stage rotors; therefore, the first mixer chamber temperature was set to a higher temperature (Table 1).

A full factorial experiment trial was performed for processing on the continuous mixer. The variables included three processing parameters and two equipment variables. The processing parameters were the feed rate, rotor speed, and orifice opening. The low and high levels of processing parameter variables for the screening experiment are shown in Table 2. These variables were based on a maximum rotor speed of 1000 rpm and typical rotor speeds of 300–700 rpm [7], a maximum feed rate of 30 kg/h, and experience showing that orifice openings greater than 40% produce significant levels of unmelted HDPE particles. The equipment variables were the rotor designs and single-screw designs presented in Figure 3 and Figure 4, respectively.

### 2.3. Data Collection and Analysis

For all experimental trials, the continuous mixer was run with the selected parameters for 20 min prior to data collection to ensure steady state conditions. The melt temperatures were recorded from the machine’s readouts for the mixer chamber and single-screw extruder; the thermocouples are located at the discharge end of the mixer and the extruder. In addition, a separate hand-held thermocouple (Omega Engineering Inc., Norwalk, CT, USA) was used to confirm the melt temperatures for both the mixing section and the extruder. The drive torques for the mixing rotors and the extruder screw were recorded from the machine’s readouts. A pressure transducer (Dynisco, Franklin, MA, USA) was used to measure head pressure at the end of the extruder. For the mixer residence time and total residence time, a black pigment was used as a tracer to determine the residence times for the mixing chamber and the mixing chamber plus the single-screw extruder. The black pigment was fed directly into the mixing section’s feed port and residence time was measured by detecting visual changes in the color of the extrudate as it exited the mixer or the single-screw extruder. The total residence time was the travel time from feed port to die exit. To ensure accurate results, at least three replicates were conducted for each trial.

Although the mixing section was starve-fed, drag flow was the primary conveyance mechanism for the mixing section and the single-screw extruder. The drive torque for the mixing rotors decreased with increasing rotor speed because the material moved faster through the mixing section, reducing the fill ratio. This drive torque also increased with higher feed rates due to greater filling of the rotors; these trends were consistent with the specific energy results reported by Valsamis and Canedo [27]. With a constant feed rate, the torque decreased with wider orifice openings; this decrease was due to lower fill in the rotor. For all trials with the single-screw extruder, the drive torque was unchanged because the screw speed was constant. The average head pressure remained constant at about 25 bar. A constant drive torque and head pressure indicated a constant output from extruder. As expected, gravity feeding of the melt from the mixing section, a constant extruder screw speed, and the same channel depth in the metering zone of both screws permitted drag flow-controlled output of the single-screw extruder. Therefore, the critical responses were the melt temperatures and the residence times.

To understand which parameters were responsible for the variation in responses, analysis of variance analysis (ANOVA) was performed to determine whether individual and interactions impacted (1) the mixer residence time, (2) the total residence time, (3) the mixer melt temperature, and (4) the extruder melt temperature. Analyses were performed with the averaged values for residence times, but the standard errors for the data were incorporated into the statistical analysis. The stabilized melt temperatures were incorporated into the ANOVA analysis. SAS JMP^®^ 17.2 statistical software (Cary, NC, USA) was used to analyze the collected data. These data were statistically analyzed using one-way analysis of variance (ANOVA) to assess statistical significance. A 95% confidence interval was used for all the analyses. The confidence interval covers 95% of the mean data, and any values outside of the interval are considered significantly different (*p* < 0.05). The 5% error margin for the dataset provides a balance between precision and certainty; this threshold is commonly set by researchers and manufacturing. After regression analysis, all terms term with *p* > 0.05 were eliminated from the regression model, and the data were reanalyzed again to improve the accuracy of the statistical analysis reported in Section 3.

## 3. Results and Discussion

Table 3 and Table 4 present a summary of the variables for the 80 experimental trials and their corresponding average mixer residence times (t_mixer_), average total residence times (t_total_), mixer melt temperatures (T_mixer_), and extruder melt temperatures (T_extruder_). The results showed that mixer residence times, total residence times, and mixer melt temperatures differed across trials. The average mixer residence times and their standard deviations ranged from 6.2 s ± 0.3 s to 20.1 s ± 0.7 s (trials 15 and 74, respectively). The average total residence times varied from 50.2 s ± 1.2 s to 139.5 s ± 2.4 s (trials 15 and 65, respectively). Overall, the changes in average mixer residence time and average extruder residence time were significant. In contrast, the mixer and extruder melt temperatures for a given trial reached stable values after 20 min; these temperatures did not change during the rest of a given trial. As the processing parameters and rotor and screw designs were varied, the mixer melt temperature ranged between 156 °C and 177 °C, and the extruder melt temperature varied between 173 °C and 179 °C. Therefore, the change in variables resulted in a significant change in the mixer melt temperature (~21 °C), but a very small difference in the extruder melt temperature (~7 °C).

**Figure 5:**
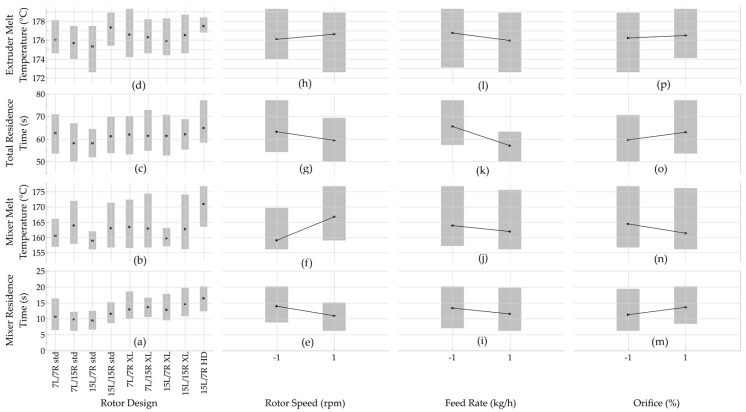
Mean values for mixer residence time, mixer melt temperature, total residence time, and extruder melt temperature as a function of (**a**–**d**) rotor design, (**e**–**h**) rotor speed, (**i**–**l**) feed rate, and (**m**–**p**) orifice opening; the “gray bands” indicate on the data ranges of each set of experiments.

As shown in Figure 5a, the mean (average) mixer residence time data indicated that rotor design affected mixer residence time trends. The mean mixer residence times for the standard rotors was 9.5 s to 11.5 s. The two-stage rotors produced longer mean mixer residence times of 12.8 s to 14.6 s, while the HD rotors provided a mean mixing time of 16.4 s. These trends were due to the polymer travel distance (i.e., rotors’ length) and the length of the mixing section. The single-stage rotors were shorter (205 mm), and the mixing section was 122 mm long. In contrast, the two-stage rotors were longer (328 mm), but they had the same mixing section length (122 mm). While the HD rotors also had a rotor length of 328 mm, the longer (201 mm) mixing produced the longest mixer residence time. The greater back flow in the longer mixing sections created longer residence times. When using single-stage or two-stage rotors, style 15 (15L/15R) rotors produced longer mixer residence times compared to style 7 rotors and to combinations of style 7 and 15 rotors. This behavior was due to location and length of apex zones. In style 15 rotors, the apex was located further upstream and was longer than in style 7 rotors; the apex length for style 7 is about 70 mm, whereas it is about 75 mm for style 15. With 7/7 and 15/15 rotors, the axial distance was 13 mm to 15 mm, and there was no offset between the tips. In contrast, the axial distance was 20 mm to 25 mm and there was no offset with 15/7 rotors; when using 7/15 rotors, the axial distance was 18–30 mm, and there was an offset between the tips. Moreover, back flow resulted in the HDPE spending more time in the reverse section after the apex zone [6,11].

In contrast, rotor design had limited effects on the mixer melt temperature (Figure 4b). The mean mixer melt temperatures were 161.6 °C and 162.2 °C for the standard and two-stage rotors, respectively, and 171 °C for the HD rotor. With single-stage rotors, the polymer melts at the beginning of the 122 mm long mixing section [28], and in two-stage rotors, melting occurs at the beginning of the 122 mm long second mixing section [12]. As a result, both single and two-stage rotors created the same opportunities for shear heating of the melt. The premixing sections of the two-stage rotors did not contribute to changes in the melt temperatures. In the HD rotors, the molten polymer was exposed to shear for longer time as it traveled along the 201 mm long mixing section. The longer mixing section also produced greater backflow. Therefore, the HD rotor presented the highest mean mixer melt temperatures.

With different processing parameters, some rotor designs exhibited tightly clustered ranges of melt temperatures, which indicated low temperature variability. Other rotor designs had a broader distribution of melt temperatures, which indicated greater variability in the mixer melt temperature. In particular, the standard rotors showed a more compact temperature distribution, whereas the two-stage rotors showed a wider spread of temperatures. This behavior was consistent with previous results for continuous mixers, where a significant portion of the energy generated when melted passed over the rotors was converted into internal energy, raising the melt temperature [6].

Figure 5c presents data for the total residence time, which was the travel time from the feed port to the die exit. The mean total residence times indicated that rotor design had no consistent impact on the total residence time. For instance, the single-stage and two-stage style 7 and style 15 rotors showed comparable total residence times of 58 s to 62 s. The HD rotors exhibited a slight increase in mean total residence time (65 s) when compared to the other rotors. This trend was consistent with the mixer residence time results and was due to the longer axial length of the two-stage and HD rotors. The extruder melt temperature was also not significantly influenced by rotor design (Figure 5d). The mean melt temperatures across different rotor designs exhibited no clear trend. This behavior could be because the role of the single-screw extruder in the continuous mixer is to pump the polymer melt, which had already been melted in the mixer section.

As expected, the mean mixer residence time decreased when the rotor speed was increased from 400 rpm to 800 rpm (Figure 5e); the higher rotor speed moved the material more rapidly through the mixing section. The same behavior was observed with an increase in feed rate from 15 kg/h to 20 kg/h (Figure 5i); higher feed rates produce greater levels of fill, which allowed the HDPE to travel faster through the mixing section. These observations were consistent with trends reported for polypropylene processed with style 15 rotors [11]. When the orifice opening was increased, however, the residence time increased due to melt quality changes (Figure 5m). This observation differed from work with polypropylene, where increasing the orifice opening reduced the residence time due to lower back flow (i.e., the melt exited the mixer faster) [11]. This difference in trends may have been due to the mixer size; the mixer used by Galle and White was twice the size of the mixer used in this study [11]. Since the mixer size can affect residence time and melt quality, Galle and White were able to achieve an excellent melting quality and see a clear reduction in mixer residence time.

The reduction in mixer residence time did affect the melt temperature measured in the mixer. While the faster rotor speed reduced the residence time, it also increased the level of shear in the mixer; the result was a higher melt temperature (Figure 5f). The greater feed rate increased the level of fill, thereby causing less shearing and a reduction in the melt temperature (Figure 5j). While the larger orifice opening increased the residence time, it also allowed the rotors to impart less shear to the HDPE. The result was a decrease in mixer melt temperature (Figure 5n). Again, the results for rotor speed and feed were consistent with those previous reported for polypropylene but changing the level of orifice closing provided the oppositive trend [11].

The total residence time decreased with increases in rotor speed and feed rate (Figure 5g and Figure 5k, respectively), but it increased with larger orifice openings (Figure 5o). Higher rotor speeds and the greater levels of fill associated with higher feed rates allowed the HDPE to travel more rapidly through the mixer. Since the single-screw extruder (SS1) did not restrict flow, it could pump the additional melt and therefore reduce the total residence time. As discussed earlier, the larger orifice opening increased the mixer residence time. This increase in residence time produced an increase in the total residence time for the mixer and extruder. Comparable results had not been reported in prior studies of continuous mixers.

As illustrated in Figure 5h,l,p, the extruder melt temperature increased with higher rotor speeds and greater orifice openings, but it decreased with higher feed rates. The higher rotor speeds imparted more shear to the HDPE, while the greater orifice openings increased the residence time in the mixer. In contrast, the greater feed rates reduced the shearing of the HDPE in the mixer. Again, results for this parameter had not been reported in prior studies with continuous mixers.

**Figure 6:**
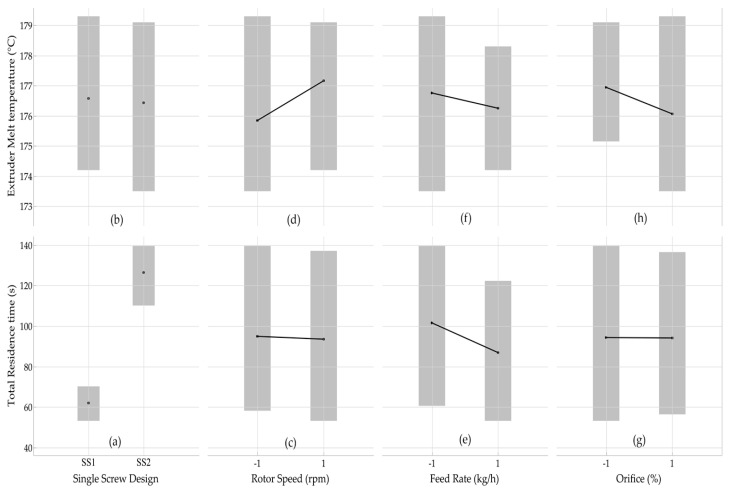
Mean values of total residence time and extruder melt temperature as a function of (**a**,**b**) single-screw design, (**c**,**d**) rotor speed, (**e**,**f**) feed rate, and (**g**,**h**) orifice opening. The “gray bands” are based on the data ranges of each set of experiments.

Figure 6 illustrates the effect of the single-screw design on the total residence time and extruder melt temperature at varying rotor speed, feed rate, and orifice opening using style 7L/7R XL rotors. The results indicated that the single-screw design and feed rate had a greater impact on the total residence time than the rotor speed and orifice opening. In the case of a single-screw design (Figure 5a), the mean data showed clear trends, indicating that the single-screw design had a significant impact on the total residence time. The mean total residence time of the single-screw extruder design with a CR of 1:1 increased from 62 s to 126 s. As a result, more time was required to fill the screw channel. Similarly, a higher feed rate (Figure 6e) corresponded to a lower total residence time, indicating that these parameters could play a significant role in determining the total residence time. On the other hand, the mean data for rotor speed (Figure 6c) and orifice opening (Figure 6g) did not show a clear trend, indicating that rotor speed and orifice opening alone did not significantly influence total residence time. Figure 6b,d,f,h present the effects of four operational parameters on extruder melt temperature—i.e., screw design, rotor speed, feed rate, and orifice opening. The screw design, rotor speed, feed rate, and orifice opening, however, did not significantly influence melt temperature.

The ANOVA analysis results in Table 5 show the significant effects of the main factors on mixer residence time and their significant interactions. As indicated by an F ratio of 11.5 and a *p*-value less than 0.0001, the overall model was statistically significant. These results suggested that the main factors and combinations of these factors have a significant effect on the mixer residence time. The intercept of the model had an estimate of 12.42 (standard error = 0.31) and a *p*-value of < 0.0001, indicating that the baseline level of mixer residence time was approximately 12.42 units when all other factors were at their reference levels.

The data for the 15L/15R standard rotor were the reference for the ANOVA analysis. As presented in Table 5, the HD rotor (15L/7R HD) showed a significant positive effect on residence time, with an estimate of 3.99 (standard error = 0.42) and *p*-value of <0.0001. Thus, this design led to a higher residence time than the reference design. The two-stage rotor designs 15L/15R XL and 7L/15R XL also had significant *p*-values (<0.0001 and 0.0072, respectively) and positive estimate values of 2.18 (standard error = 0.42) and 1.19 (standard error = 0.42), respectively. The estimate values indicated that those rotor designs led to a higher mixer residence times than the standard rotors, but lower residence times that produced by the HD rotor. In contrast, the single-stage rotor designs 15L/7R std, 7L/15R std, and 7L/7R std had negative estimates of −2.96, −2.58, and −1.80, respectively; all had SEs = 0.42 and *p*-values of 0.0001. The negative estimates indicated these rotor designs reduced the mixer residence time in comparison with the reference design. Rotors showing *p*-values > 0.05 probably had mixer residence times similar to that of the reference rotor (15L/15R). If the highest mixer residence time rotor design was based on the estimate values, the order for the rotors was as follows:

15L/7R HD >> 15L/15R XL > 7L/15R XL >> 7L/7R XL ≈ 15L/7R XL ≈ 15L/15R std > 7L/7R std > 7L/15R std > 15L/7R std.

All three processing variables, the rotor speed, feed rate, and orifice opening, exhibited significant *p*-values of <0.0001, 0.0.0058, and 0.0008, respectively. Rotor speed and feed rate had negative estimates of −1.50 and −0.92, respectively, which were consistent with the tendency of these parameters to reduce mixer residence time. The orifice opening, however, had a positive effect, with an estimate of 1.15 (standard error = 0.31), which indicated an increase in residence time with a larger orifice opening.

Based on *p*-values of >0.05, most interactions between the variables had negligible effects on mixer residence time. The interaction between the HD rotor design (15L/7R HD) and rotor speed, however, had a significant negative effect with an estimate of –1.21 (standard error = 0.42) and *p*-value of 0.0064. These results indicated that increasing rotor speed caused a −1.21-s reduction in mixer residence time. In addition, the interaction between rotor speed and orifice opening showed a significant effect with a *p*-value of 0.0255 and an estimate of −0.33 (standard error = 0.14). While statistically significant, this interaction was practically insignificant because it reduced the residence time by only 0.33 s.

Table 6 presents the ANOVA analysis for the total residence time. As indicated by the F ratio of 217.4, the overall model was statistically significant, with a *p*-value less than 0.0001. The model’s intercept had an estimate of 93.53 with a standard error of 0.48, yielding a highly significant *p*-value (*p* < 0.0001). With total residence time, the dominant factors were the single-screw design, the feed rate, and some rotor designs; the rotor speed and orifice opening did not exhibit significant effects. The single-screw design showed a strong positive effect on total residence time with an estimate of 32.18 (standard error = 0.48) and *p*-value of 0.0001. Thus, a substantial increase in total residence time occurred when using single screw with compression ratio of 1:1. The feed rate, however, exhibited a negative effect on total residence time with an estimate of −7.32 (standard error = 0.48) and *p*-value of 0.0001. Thus, an increase in feed rate resulted in a reduction in the total residence time. In contrast, the rotor designs had mixed impacts on total residence time. The single-stage rotor designs 15L/7R std and 7L/15R std showed negative effects on total residence time with estimates of –3.22 (standard error = 0.64) and −3.19 (standard error = 0.64), respectively, and *p*-values lower than 0.0001 for both rotor designs. The results were consistent with the ANOVA analysis for the mixer residence time and suggested that shorter residence time in the mixer significantly influenced the shorter total residence time (compared to the reference rotor design). The HD rotor resign (15L/7R HD) had a positive impact on total residence time with an estimate of 3.55 (standard error = 0.64) and *p*-value < 0.0001. The single-stage rotor design 7L/7R std exhibited the same trend, but its significance was marginal; i.e., the *p*-value was 0.452. As a result, these rotor designs contributed to longer residence times.

Additionally, the interactions of the variables showed some significant impacts (Table 6). The interactions of single-screw design with the processing variables of feed rate, orifice opening, and rotor speed showed *p*-values of <0.0001, <0.0001, and 0.0040, respectively. Estimates of −3.07 (standard error = 0.48) and −2.57 (standard error = 0.48), respectively, suggested that the greater amounts of melt fed to the single-screw extruder with higher feed rates and greater orifice openings produced reductions in the total residence time. In contrast, the interaction of screw speed and rotor speed showed an estimate of 1.47 (standard error = 0.48), indicating a smaller reduction in performance. The interactions of the rotor designs and processing variables and the processing variables were not as significant since the *p*-values varied from 0.0092 to 0.0403. Overall, the ANOVA analysis indicated that the total residence time was significantly affected by single-screw design, feed rate, and specific rotor designs, with significant interactions to be considered when optimizing the total residence time.

The ANOVA analysis shown in Table 7 assessed the effects of rotor speed, orifice size, feed rate, and rotor design configurations on mixer melt temperature. The overall model was highly significant, as indicated by an F ratio of 29.9 with a *p*-value of less than 0.0001. The model intercept had an estimate of 162.91 and a *p*-value of <0.0001. These values indicated a baseline mixer melt temperature of approximately 162.91 units when all other factors were at their reference levels.

Some rotor designs had a significant impact on the mixer melt temperature (Table 7). The HD rotor design (15L/7R HD) had a positive estimate of 8.08 (standard error = 0.48) and *p*-value of < 0.0001, indicating an increase in melt temperature compared to the reference rotor design. This result was not expected given the longer mixing section on the HD rotor design and the higher set temperature at the mixer’s entrance. The single-stage rotor design 7L/15R std exhibited a smaller positive estimate of 1.00 (standard error = 0.48), but it was not as significant (*p*-value = 0.0445). In contrast, rotor designs 15L/7R std, 15L/7R XL, and 7L/7R std had negative estimates of −3.98, −3.16, and −2.40, respectively, and all had *p*-values of < 0.0001. These results suggested a decrease in melt temperature compared to the reference design. The results were consistent with the designs in that rotor design 7L/7R std had been designed to provide less shearing and the 15L/7R std and 15L/7R XL combinations provide faster movement and less shearing of the material. Based on the estimate values for rotor design only, the order of rotor design that produced the highest rise in mixer melt temperature was as follows:

15L/7R HD >> 7L/15R std > 7L/7R XL > 15L/15R std ≈ 7L/15R XL ≈ 15L/15R XL > 7L/7R std > 15L/7R XL > 15L/7R std.

As shown in Table 7, the rotor speed had a significant positive effect on mixer melt temperature, with an estimate of 3.87 (standard error = 0.48) and a *p*-value of < 0.0001. These values suggested that an increase in rotor speed was associated with a substantial increase in mixer melt temperature. In contrast, orifice opening had a significant negative effect, with an estimate of −1.53 (standard error = 0.36) and a *p*-value of 0.0001, suggesting that increasing the orifice opening leads to a decrease in mixer melt temperature. Greater feed rates, which increase the fill in the mixer, produced a smaller decrease in melt temperature. The estimate was −0.97 (standard error = 0.36) with a *p*-value of 0.0108.

The interactions between two-stage rotor designs 7L/15R XL and 15L/7R XL and orifice opening were significant with *p*-values of <0.0001 and 0.0003, respectively. The estimate for rotor design 7L/15R XL was −2.63 (standard error = 0.48), which indicated that larger orifice openings reduced the mixer melt temperature. Since the estimate for rotor design 15L/7R XL was −1.93 (standard error = 0.48), larger orifice openings increased the mixer melt temperature. In addition, the interaction between rotor speed and orifice opening had an estimate of −0.74 (standard error = 0.16) and *p*-value of <0.0001, indicating that the combined effect of rotor speed and orifice size reduces the melt temperature. As shown in Table 7, the interactions between rotor designs and rotor speed produced small changes in mixer melt temperature. For the two-stage rotor designs 15L/7R XL, 15L/15R XL, and 7L/7R XL as well as the HD rotor design (15L/7R HD), the estimates of 1.10 to 1.93 (standard error = 0.48) indicated an increase in melt temperature. With the single-stage rotor designs 15L/7R std and 7L/7R std and the two-stage rotor design 15L/5R XL, the estimates of −1.38 to −1.89 (standard error = 0.48) suggested similar small decreases in mixer melt temperature.

Table 8 presents the results of ANOVA analysis conducted to assess the significance of the model on extruder melt temperature. The calculated F-ratio for the model was 2.1, with a corresponding *p*-value of 0.0136. Since the *p*-value was below the threshold of 0.05, the model was deemed statistically significant. The intercept had an estimate of 176.28 with a standard error of 0.30, resulting in a highly significant *p*-value of < 0.0001.

As shown in Table 8, only limited factors impacted the melt temperature measured at the extruder exit. The rotor speed exhibited an estimate of 0.90 (standard error = 0.30) and a significant *p*-value of 0.0042, indicating that greater rotor speeds produced a small increase in extruder melt temperature. Two rotor designs also had some effect on extruder melt temperature. The HD rotor design (15L/7R HD) had a positive estimate of 1.13 (standard error = 0.39) with a significant *p*-value of 0.0069. This result suggested that using the HD-rotor design would cause an increase in extruder melt temperature compared to using the standard rotor design (15L/15R std). In contrast, the single-stage rotor design 15L/7R std showed an estimate of −1.03 (standard error = 0.39) and a *p*-value of 0.0133, which indicated a reduction in extruder melt temperature.

The interaction between factors showed some significance. First, the interaction between feed rate and orifice opening had an estimate of −0.52 (standard error = 0.13) and a *p*-value of 0.0003, indicating strong significance, although the change in temperature was not large. This result was not unexpected because both factors influence the rate at which polymer flows from the mixer to the extruder. Second, the interaction between rotor design 15L/7R std and rotor speed had an estimate of −0.97 (standard error = 0.39) and *p*-value of 0.0188. Third, the interaction between screw design SS2 and rotor speed had a positive estimate of 0.63 and a *p*-value of 0.0388, indicating that the extruder melt temperature for the screw with a compression ratio of 1:1 was slightly higher with faster rotor speeds. Overall, the ANOVA results for extruder melt temperature suggested that the melt temperature changed less than 1 °C with changes in rotor design, rotor speed, feed rate, and orifice opening. Moreover, high levels of viscous dissipation did not occur in the single-screw extruder.

## 4. Conclusions

Continuous mixers are widely utilized in melt compounding processes, particularly for incorporating high levels of additives into thermoplastics for color masterbatches, and highly filled compounds. These mixers offer distinct advantages over melt compounding techniques, including lower processing temperatures, reduced mechanical shear, and decreased specific energy requirements, making them suitable for processing recycled and heat-sensitive materials. The current applications, however, generally utilize only one rotor design. This study investigated the impact of nine rotor designs and two single-screw designs on the residence time in the mixer, the overall residence time, the melt temperature at the end of the mixer, and the melt temperature at the end of the extruder using high density polyethylene resin. This evaluation was performed as a factorial design with five parameters (rotor design, screw design, rotor speed, feed rate, and orifice position) and an ANOVA analysis. Results showed that the single-screw design with the 1:1 compression ratio significantly increased the total residence time due to the lower channel volume compared to the single screw standard compression ratio of 2.3:1. This change in screw design did not impact melt temperature measured at the end of the extruder. The single-stage (standard) rotors generally produced lower mixer residence times and mixer melt temperatures compared to the two-stage and high dispersion (HD) rotors. The rotor designs alone, however, did not significantly impact the residence time and melt temperature. Greater rotor speeds and feed rates and smaller orifice openings generally produced reductions in mixer residence time. Higher rotor speeds increased mixer melt temperature, whereas higher feed rates and smaller orifice openings produced lower mixer melt temperature. Overall, higher shear levels in the mixer resulted in a higher melt temperature, whereas the higher fill levels associated with greater feed rates resulted in less shearing and a reduction in the melt temperature. The trends from the mixers could often be observed in the total residence time and extruder melt temperature. The results of this work provide strategies for optimizing rotor design selection and processing parameters when processing recycled resins, compounding heat-sensitive materials (such as PLA and PET), or performing reactive extrusion with a continuous mixer.

## Figures and Tables

**Table 1:** Set temperatures for the continuous mixer trials.

	Std and Two Stage Rotors	HD Rotor
Mixer Chamber 1 (°C)	65	95
Mixer Chamber 2 (°C)	165	165
Orifice (°C)	165	165
Extruder Cylinder 1 (°C)	170	170
Extruder Cylinder 2 (°C)	170	170
Die (°C)	170	170

**Table 2:** Low/high levels for processing variables for the screening experiments.

Factors	Rotor Speed (rpm)	Feed Rate (kg/h)	Orifice Opening (%)
Low Level (−)	400	15	20
High Level (+)	800	20	40

**Table 3:** Overview of processing conditions with the corresponding mixer residence time, total residence time, mixer melt temperature, and extruder melt temperature for the single-stage rotors and the HD rotor.

Trial	Screw Design	Rotor Design	Rotor Speed	Feed Rate	Orifice %	t_mixer_ (s)	t_total_ (s)	T_mixer_ (°C)	T_extruder_ (°C)
1	SS1	7L/7R std	−1	−1	−1	11.2	66.0	157.7	174.9
2	SS1	7L/7R std	−1	−1	1	16.4	71.0	158.2	178.1
3	SS1	7L/7R std	−1	1	−1	9.4	55.1	159.3	175.0
4	SS1	7L/7R std	−1	1	1	11.5	61.9	156.9	174.6
5	SS1	7L/7R std	1	−1	−1	9.3	65.6	166.1	176.4
6	SS1	7L/7R std	1	−1	1	11.5	69.4	161.1	177.5
7	SS1	7L/7R std	1	1	−1	6.5	53.6	164.6	176.6
8	SS1	7L/7R std	1	1	1	9.3	58.8	160.2	175.0
9	SS1	7L/15R std	−1	−1	−1	12.0	62.5	158.5	174.0
10	SS1	7L/15R std	−1	−1	1	12.2	67.0	159.4	175.4
11	SS1	7L/15R std	−1	1	−1	10.7	55.1	161.5	175.9
12	SS1	7L/15R std	−1	1	1	8.8	55.1	157.9	175.0
13	SS1	7L/15R std	1	−1	−1	8.1	59.1	172.0	175.6
14	SS1	7L/15R std	1	−1	1	11.5	60.4	166.5	177.4
15	SS1	7L/15R std	1	1	−1	6.2	50.2	170.5	177.5
16	SS1	7L/15R std	1	1	1	9.2	55.9	165.0	174.7
17	SS1	15L/7R std	−1	−1	−1	10.8	61.5	157.5	175.8
18	SS1	15L/7R std	−1	−1	1	12.5	64.5	157.2	177.5
19	SS1	15L/7R std	−1	1	−1	9.0	54.3	157.0	176.7
20	SS1	15L/7R std	−1	1	1	11.6	60.1	156.1	174.1
21	SS1	15L/7R std	1	−1	−1	7.0	57.4	162.0	173.1
22	SS1	15L/7R std	1	−1	1	9.8	61.8	160.8	175.8
23	SS1	15L/7R std	1	1	−1	6.6	52.0	161.8	172.6
24	SS1	15L/7R std	1	1	1	8.4	53.6	159.0	177.0
25	SS1	15L/15R std	−1	−1	−1	11.6	66.5	161.9	176.7
26	SS1	15L/15R std	−1	−1	1	15.2	69.9	158.2	177.5
27	SS1	15L/15R std	−1	1	−1	10.8	55.1	158.7	177.0
28	SS1	15L/15R std	−1	1	1	12.8	59.0	156.7	177.1
29	SS1	15L/15R std	1	−1	−1	10.5	61.5	171.4	178.4
30	SS1	15L/15R std	1	−1	1	12.5	68.3	167.0	177.7
31	SS1	15L/15R std	1	1	−1	8.6	53.8	168.5	178.9
32	SS1	15L/15R std	1	1	1	10.4	56.4	161.7	175.4
73	SS1	15L/7R HD	−1	−1	−1	19.4	70.6	169.7	177.1
74	SS1	15L/7R HD	−1	−1	1	20.1	77.2	166.5	177.6
75	SS1	15L/7R HD	−1	1	−1	17.3	63.3	164.4	176.8
76	SS1	15L/7R HD	−1	1	1	19.7	62.3	163.5	177.0
77	SS1	15L/7R HD	1	−1	−1	14.7	63.4	176.8	177.5
78	SS1	15L/7R HD	1	−1	1	15.1	63.2	176.2	178.3
79	SS1	15L/7R HD	1	1	−1	12.3	58.4	175.6	178.4
80	SS1	15L/7R HD	1	1	1	12.7	60.8	175.2	177.2

**Table 4:** Overview of processing conditions with the corresponding mixer residence time, total residence time, mixer melt temperature, and extruder melt temperature for the two-stage rotors.

Trial	Screw Design	Rotor Design	Rotor Speed	Feed Rate	Orifice %	t_mixer_ (s)	t_total_ (s)	T_mixer_ (°C)	T_extruder_ (°C)
33	SS1	7L/7R XL	−1	−1	−1	12.6	66.2	160.9	175.8
34	SS1	7L/7R XL	−1	−1	1	18.6	70.2	157.8	179.3
35	SS1	7L/7R XL	−1	1	−1	13.6	58.2	158.3	175.8
36	SS1	7L/7R XL	−1	1	1	15.9	63.2	156.5	175.3
37	SS1	7L/7R XL	1	−1	−1	10.1	60.6	172.4	177.7
38	SS1	7L/7R XL	1	−1	1	11.8	68.1	167.5	176.7
39	SS1	7L/7R XL	1	1	−1	9.9	53.2	169.9	177.8
40	SS1	7L/7R XL	1	1	1	10.9	56.4	164.0	174.2
41	SS1	7L/15R XL	−1	−1	−1	14.4	65.7	163.5	176.3
42	SS1	7L/15R XL	−1	−1	1	16.6	72.9	157.7	176.8
43	SS1	7L/15R XL	−1	1	−1	12.5	58.5	158.2	174.8
44	SS1	7L/15R XL	−1	1	1	14.5	57.1	156.7	174.9
45	SS1	7L/15R XL	1	−1	−1	14.6	63.3	174.4	176.6
46	SS1	7L/15R XL	1	−1	1	14.2	62.3	161.3	178.2
47	SS1	7L/15R XL	1	1	−1	10.5	54.8	172.3	178.2
48	SS1	7L/15R XL	1	1	1	11.7	56.8	159.4	174.6
49	SS1	15L/7R XL	−1	−1	−1	12.6	62.1	157.3	174.9
50	SS1	15L/7R XL	−1	−1	1	17.8	70.8	158.1	176.7
51	SS1	15L/7R XL	−1	1	−1	11.6	57.6	157.3	176.8
52	SS1	15L/7R XL	−1	1	1	13.7	60.7	157.1	174.8
53	SS1	15L/7R XL	1	−1	−1	10.5	61.0	162.1	174.8
54	SS1	15L/7R XL	1	−1	1	13.4	66.8	163.1	178.3
55	SS1	15L/7R XL	1	1	−1	9.5	52.7	160.7	174.4
56	SS1	15L/7R XL	1	1	1	13.3	59.7	162.3	176.7
57	SS1	15L/15R XL	−1	−1	−1	14.8	67.5	158.6	175.8
58	SS1	15L/15R XL	−1	−1	1	19.7	68.8	157.9	177.5
59	SS1	15L/15R XL	−1	1	−1	10.8	61.4	156.7	174.6
60	SS1	15L/15R XL	−1	1	1	19.0	58.5	156.1	175.1
61	SS1	15L/15R XL	1	−1	−1	14.8	64.5	174.1	176.7
62	SS1	15L/15R XL	1	−1	1	12.8	64.0	168.3	178.7
63	SS1	15L/15R XL	1	1	−1	11.4	55.4	167.6	178.0
64	SS1	15L/15R XL	1	1	1	13.6	57.0	162.5	175.8
65	SS2	7L/7R XL	−1	−1	−1	12.6	139.5	160.9	175.2
66	SS2	7L/7R XL	−1	−1	1	18.6	136.4	157.8	173.5
67	SS2	7L/7R XL	−1	1	−1	13.6	117.4	158.3	175.9
68	SS2	7L/7R XL	−1	1	1	15.9	110.1	156.5	176.0
69	SS2	7L/7R XL	1	−1	−1	10.1	137.0	172.4	179.1
70	SS2	7L/7R XL	1	−1	1	11.8	134.1	167.5	176.8
71	SS2	7L/7R XL	1	1	−1	9.9	122.3	169.9	178.3
72	SS2	7L/7R XL	1	1	1	10.9	114.1	164.0	176.7

**Table 5:** ANOVA analysis for mixer residence time.

Analysis of Variance					
**Source**	**Sum of Squares**	**DoF**	**Mean Square**	**F Ratio**	***p*-Value**
Model	766.6	42	18.3	11.5	<0.0001
Error	58.6	37	1.6		
Total	825.2	79			
**Term**		**Estimate**	**Std Error**	**t Ratio**	***p*-Value**
Intercept		12.42	0.31	39.5	<0.0001
Rotor Design [15L/7R HD]		3.99	0.42	9.51	<0.0001
Rotor Design [15L/7R std]		−2.96	0.42	−7.07	<0.0001
Rotor Design [7L/15R std]		−2.58	0.42	−6.15	<0.0001
Rotor Design [15L/15R XL]		2.18	0.42	5.19	<0.0001
Rotor Design [7L/7R std]		−1.80	0.42	−4.29	0.0001
Rotor Design [7L/15R XL]		1.19	0.42	2.85	0.0072
Rotor Speed		−1.50	0.31	−4.78	<0.0001
Orifice Opening		1.15	0.31	3.65	0.0008
Feed Rate		−0.92	0.31	−2.93	0.0058
Rotor Design [15L/7R HD] × Rotor Speed		−1.21	0.42	−2.89	0.0064
Rotor Speed × Orifice Opening		−0.33	0.14	−2.33	0.0255

**Table 6:** ANOVA analysis for total residence time.

Analysis of Variance					
**Source**	**Sum of Squares**	**DoF**	**Mean Square**	**F Ratio**	***p*-Value**
Model	33,578.2	42	799.5	217.4	<0.0001
Error	136.1	37	3.7		
Total	33,714.2	79			
**Term**		**Estimate**	**Std Error**	**t Ratio**	***p*-Value**
Intercept		93.53	0.48	195.1	<0.0001
Screw Design [SS2]		32.18	0.48	67.12	<0.0001
Rotor Design [15L/7R HD]		3.55	0.64	5.56	<0.0001
Rotor Design [15L/7R std]		−3.22	0.64	−5.03	<0.0001
Rotor Design [7L/15R std]		−3.19	0.64	−4.98	<0.0001
Rotor Design [7L/7R std]		1.32	0.64	2.07	0.0452
Feed Rate		−7.32	0.48	−15.26	<0.0001
Screw Design [SS2] × Feed Rate		−3.07	0.48	−6.4	<0.0001
Screw Design [SS2] × Orifice		−2.57	0.48	−5.37	<0.0001
Screw Design [SS2] × Rotor Speed		1.47	0.48	3.07	0.0040
Rotor Design [15L/15R XL] × Orifice		−1.76	0.64	−2.75	0.0092
Rotor Design [15L/7R HD] × Rotor Speed		−1.55	0.64	−2.43	0.0200
Rotor Design [15L/7R XL] × Orifice		1.38	0.64	2.15	0.0378
Rotor Speed × Feed Rate		0.46	0.21	2.13	0.0403

**Table 7:** ANOVA analysis for mixer melt temperature.

Analysis of Variance					
**Source**	**Sum of Squares**	**DoF**	**Mean Square**	**F Ratio**	***p*-Value**
Model	2631.4	42	62.7	29.9	<0.0001
Error	77.6	37	2.1		
Total	2709.0	79			
**Term**		**Estimate**	**Std Error**	**t Ratio**	***p*-Value**
Intercept		162.91	0.36	449.92	<0.0001
Rotor Design [15L/7R HD]		8.08	0.48	16.73	<0.0001
Rotor Design [15L/7R std]		−3.98	0.48	−8.25	<0.0001
Rotor Design [15L/7R XL]		−3.16	0.48	−6.54	<0.0001
Rotor Design [7L/7R std]		−2.40	0.48	−4.96	<0.0001
Rotor Design [7L/15R std]		1.00	0.48	2.08	0.0445
Rotor Speed		3.87	0.36	10.68	<0.0001
Orifice Opening		−1.53	0.36	−4.23	0.0001
Feed Rate		−0.97	0.36	−2.69	0.0108
Rotor Design [7L/15R XL] × Orifice Opening		−2.63	0.48	−5.45	<0.0001
Rotor Speed × Orifice Opening		−0.74	0.16	−4.56	<0.0001
Rotor Design [15L/7R XL] × Orifice Opening		1.93	0.48	4.00	0.0003
Rotor Design [15L/7R std] × Rotor Speed		−1.89	0.48	−3.92	0.0004
Rotor Design [15L/7R XL] × Rotor Speed		−1.57	0.48	−3.25	0.0025
Rotor Design [15L/15R XL] × Rotor Speed		1.53	0.48	3.18	0.0030
Rotor Design [7L/7R std] × Rotor Speed		−1.38	0.48	−2.86	0.0070
Rotor Design [7L/7R XL] × Rotor Speed		1.17	0.48	2.43	0.0203
Rotor Design [15L/7R HD] × Rotor Speed		1.10	0.48	2.27	0.0291
Rotor Design [15L/15R XL] × Feed Rate		−1.03	0.48	−2.13	0.0400

**Table 8:** ANOVA analysis for extruder melt temperature.

Analysis of Variance					
**Source**	**Sum of Squares**	**DoF**	**Mean Square**	**F Ratio**	***p*-Value**
Model	121.5	42	2.9	2.1	0.0136
Error	51.9	37	1.4		
Total	173.4	79			
**Term**		**Estimate**	**Std Error**	**t Ratio**	***p*-Value**
Intercept		176.28	0.30	595.42	<0.0001
Rotor Design [15L/7R HD]		1.13	0.39	2.86	0.0069
Rotor Design [15L/7R std]		−1.03	0.39	−2.60	0.0133
Rotor Speed		0.90	0.30	3.06	0.0042
Feed Rate × Orifice		−0.52	0.13	−3.95	0.0003
Rotor Design [15L/7R std] × Rotor Speed		−0.97	0.39	−2.46	0.0188
Screw Design [SS2] × Rotor Speed		0.63	0.30	2.14	0.0388

## Data Availability

Data are contained within the article.

## References

[1] Kohlgrüber K., Bierdel M., Rust H. (2021). Plastics Compounding and Polymer Processing.

[2] Martin C. (2016). Twin Screw Extruders as Continuous Mixers for Thermal Processing: A Technical and Historical Perspective. AAPS PharmSciTech.

[3] Aldhafeeri T., Alotaibi M., Barry C.F. (2022). Impact of Melt Processing Conditions on the Degradation of Polylactic Acid. Polymers.

[4] Alotaibi M., Aldhafeeri T., Barry C. (2022). The Impact of Reprocessing with a Quad Screw Extruder on the Degradation of Polypropylene. Polymers.

[5] Zhu X.Z., Yuan H.Q., Wang W.Q. (2009). Numerical Simulation of Flow Characteristics in New Co-Rotating Triangle Arrayed Triple Screw Extruders. J. Mater. Process Technol..

[6] Canedo E.L., Valsamis L.N., Manas-Zloczower I. (2009). Mixing in the Farrel Continuous Mixer. Mixing and Compounding of Polymers.

[7] Lahmann P.M., Knowlton N. (2019). The Benefits of Farrel Continuous Mixing (FCM^TM^) Technology in Processing Polylactide (PLA) Compounds. ANTEC^®^ 2019 PAPERS.

[8] Zhu L., Pan Y., Tian X., Liu H., Bian H., Wang C. (2019). Continuous Preparation and Properties of Silica/Rubber Composite Using Serial Modular Mixing. Materials.

[9] White J.L., Kim E.K. (2010). Technology of Continuous Mixers. Twin Screw Extrusion: Technology and Principles.

[10] Tadmor Z., Gogos C.G. (2006). Principles of Polymer Processing.

[11] Galle F.N., White J.L. (1999). Characterization of the Behavior and Blending Performance of a Continuous Mixer. Int. Polym. Process..

[12] Chen X., Zhang H., Chen T., Zhao H., Ji H., Ma Y., Sha J., Xie L. (2019). The Solid-State Mixing Characteristic of Two Rotor Continuous Mixer and Its Influence on Microstructure of HDPE/CaCO_3_ Composite. Polym. Compos..

[13] Chang D., White J.L. (2003). Experimental Study of Maleation of Polypropylene in Various Twin-Screw Extruder Systems. J. Appl. Polym. Sci..

[14] Shon K., Bumm S.H., White J.L. (2008). A Comparative Study of Dispersing a Polyamide 6 into a Polypropylene Melt in a Buss Kneader, Continuous Mixer, and Modular Intermeshing Corotating and Counter-Rotating Twin Screw Extruders. Polym. Eng. Sci..

[15] Shon K. (2003). Mixing Studies in Modular Intermeshing Co-Rotating Twin Screw Extruder, Modular Counter-Rotating Twin Screw Extruder, Buss Kneader, and Kobelco Nex-T Continuous Mixer. Ph.D. Dissertation.

[16] Barletta M., Genovesi A., Desole M.P., Gisario A. (2024). Melt Processing of Biodegradable Poly(Butylene Succinate) (PBS)—A Critical Review. Clean. Technol. Environ. Policy.

[17] Velghe I., Buffel B., Cardinaels R., Vandeginste V., Thielemans W., Desplentere F. (2024). Quantification of PLA Degradation in the Melt Phase Using a Parallel Plate Rheometer. Polym. Test..

[18] Velghe I., Buffel B., Vandeginste V., Thielemans W., Desplentere F. (2023). Review on the Degradation of Poly(Lactic Acid) during Melt Processing. Polymers.

[19] De Mélo T.J.A., Pinheiro L.A., Canevarolo S.V. (2010). Factorial Design to Quantify the Influence of Extrusion Parameters in the Mean Residence Time. Polímeros.

[20] Kosmalska D., Janczak K., Raszkowska-Kaczor A., Stasiek A., Ligor T. (2022). Polylactide as a Substitute for Conventional Polymers—Biopolymer Processing under Varying Extrusion Conditions. Environments.

[21] Abeykoon C., Kelly A.L., Brown E.C., Coates P.D. (2016). The Effect of Materials, Process Settings and Screw Geometry on Energy Consumption and Melt Temperature in Single Screw Extrusion. Appl. Energy.

[22] Velghe I., Buffel B., Vandeginste V., Thielemans W., Desplentere F. (2024). Effect of Melt Processing Conditions on the Degradation of PLA During Single-Screw Extrusion. AIP Conference Proceedings.

[23] Wang J., Mao Q., Jiang N., Chen J. (2021). Effects of Injection Molding Parameters on Properties of Insert-Injection Molded Polypropylene Single-Polymer Composites. Polymers.

[24] Bowen N., Guyer C., Rippon T., Daly M., Gao P., Galati V., Lograsso S., Johnston S., Masato D. (2024). Mechanical and Crystallization Properties of Hot Runner Injection Molded Virgin and Recycled Polypropylene. Polym. Eng. Sci..

[25] Compounding World October 2022 62. https://content.yudu.com/web/1rl19/0A1rl2p/CWOct22/html/index.html?page=62&origin=reader.

[26] Velghe I., Buffel B., Vandeginste V., Thielemans W., Desplentere F. (2025). Effect of Processing Conditions on Process-Induced Degradation of Poly(Lactic Acid) During Single-Screw Extrusion. J. Appl. Polym. Sci..

[27] Valsamis L.N., Canedo E.L. (1989). Mixing, Devolatilization, and Reactive Processing in the a Farrel Continuous Mixer. Int. Polym. Process..

[28] Tadmor Z., Klein I. (1970). Engineering Principles of Plasticating Extrusion.

